# Use of stable isotope ratio analysis to investigate the biology and clinical significance of seal parasites

**DOI:** 10.1017/S003118202400074X

**Published:** 2024-06

**Authors:** Annetta Zintl, Michelle Imlau, Jacklyn Schertzer, Han Zhang, Audrey Saint-Marc, Olaf Schmidt, Oscar Toomey, Hanne Jahns

**Affiliations:** 1 UCD School of Veterinary Medicine, University College Dublin, Dublin, Ireland; 2 UCD School of Agriculture and Food Science, University College Dublin, Dublin, Ireland; 3 School of Engineering, Trinity College Dublin, Dublin, Ireland

**Keywords:** *Corynosoma strumosum*, *Halarachne halichoeri*, *Otostrongylus circumlitus*, *Pseudoterranova decipiens*, stable isotope ratio analysis, trophic discrimination factor

## Abstract

Stranded seals are often infected with a broad range of parasites, although whether they are the cause of significant morbidity or an incidental finding is usually unclear. In this study we used stable isotope ratio analysis, a method frequently used to investigate food webs, to explore the extent to which common seal parasites feed directly on host tissue and fluids or absorb host-derived metabolites, which in turn may give an indication of their potential impact on the host's health. The trophic discrimination factor Δ^15^N for the nasal mite, *Halarachne halichoeri*, was mostly positive, ranging between −0.015 and 3.2‰ (*n* = 6), while for the Acanthocephalan worm, *Corynosoma strumosum* and the anisakid nematode, *Pseudoterranova decipiens*, Δ^15^N ranged between −4.2 and −2.0‰ (*n* = 7), and between −6.7 and −0.8‰ (*n* = 5) respectively. In the case of the lungworm, *Otostrongylus circumlitus*, Δ^15^N measured between −5.6 and 0‰ for worms collected in the stomach (*n* = 5), between −1.1 and 0.2‰ for worms collected from the heart (*n* = 3), between −0.7 and 1.9‰ for worms situated in the lungs (*n* = 4). Based on Δ^15^N, parasites could be clearly divided into those that were on a higher trophic level than their host suggesting a predator–prey-like relationship, and those that were not. It is hypothesized that Δ^15^N may be indicative of the clinical significance of parasite–host associations.

## Graphical abstract

**Figure:**
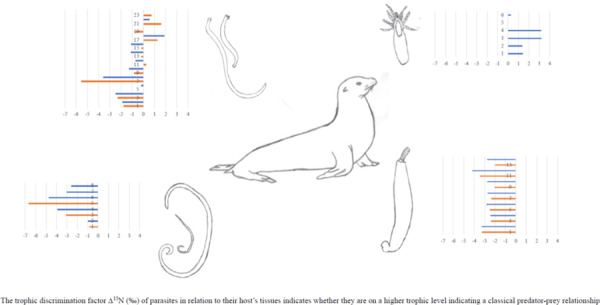

## Introduction

Stable isotope ratio analysis, a methodology conventionally used to investigate trophic relationships within food webs, is based on the fact that the tissues of animals are enriched, at a given rate, with heavy nitrogen (^15^N) and carbon (^13^C) isotopes through food consumption, assimilation and excretion. As a result, the ratios of heavy-to-light isotopes in tissues, expressed as *δ*^15^N and *δ*^13^C (‰), are specific to the individual and determined by their diet. Consumers and their food sources can be inferred by determining the difference of their respective *δ*^15^N and *δ*^13^C values, the so-called trophic discrimination factor, Δ (Post, 2002; Caut *et al*., 2009). A review of over 260 animal-diet discrimination factors reported overall mean values of 2.75‰ for Δ^15^N and 0.75‰ for Δ^13^C; however, the authors stressed that trophic discrimination factors, such as the ratios of heavy and light isotopes themselves, varied significantly between animal taxa, the environment and animal inhabits (i.e. terrestrial, freshwater or marine habitat) and the tissues that were being examined (Caut *et al*., 2009). In recent years, stable isotope ratio analysis has been employed to investigate the trophic relationships between hosts and their parasites, initially with the expectation to record trophic discrimination factors indicative of classical predator–prey relationships, i.e. a Δ^15^N of around 3.4‰ and a Δ^13^C of 0.5–1‰. However, such Δ^15^N and Δ^13^C values turned out to be the exception rather than the rule, as many parasite–host relationships are characterized by low, zero or even negative Δ^15^N and Δ^13^C values. Moreover they vary with parasite taxa, with arthropod parasites generally showing enriched Δ^15^N and Δ^13^C values in relation to their hosts, and cestodes’ depletion (Thieltges *et al*., 2019). These trophic associations are generally thought to be a reflection of the particular feeding habits and metabolic processes of parasites (Table 1) (Nachev *et al*., 2017; Thieltges *et al*., 2019; Kamiya *et al*., 2020; Riekenberg *et al*., 2021). It is likely that a better understanding of the trophic relationships between parasites and hosts will provide information regarding the parasites’ life style and the likelihood that they cause disease in their host.

**Table 1.** Proposed explanations for typical and atypical trophic discrimination factors (Δ^15^N and Δ^13^C) in host–parasite relationships

a. Δ^15^N		
High positive (~3.4‰)	Low positive (<3.4 to 0‰)	Negative (<0‰)
Direct ingestion of host tissues (resembling a predator–prey relationship)	Absorption of (^15^N depleted) host metabolites ± direct ingestion of host tissue	Absorption of ^15^N depleted metabolites produced by the host's microbiome ± absorption of the host-derived metabolites ± sharing of the host's food source

b. Δ^13^C		
Positive (1–0.5‰)	Near zero or Negative (<0‰)	
Direct ingestion of host tissues (resembling a predator–prey relationship)	Absorption and direct assimilation of lipids and fatty acids derived from the host/the host's diet	

Adapted from Riekenberg *et al*. (2021).

To our knowledge this is the first study that employs stable isotope ratio analysis to investigate the trophic relationships of grey seals (*Halichoerus grypus*) and harbour seals (*Phoca vitulina*) and their parasites. We focused on nasal mites (*Halarachne halichoeri*), Acanthocephala (*Corynosoma strumosum*) and the anisakid and metastrongyloid nematodes, *Pseudoterranova decipiens* and *Otostrongylus circumlitus* as they are some of the most common parasites of pinnipeds in the North Atlantic. *Halarachne halichoeri* inhabits the (upper) respiratory tract of its host. It has a direct life cycle and is transmitted by direct contact or sneezing. In contrast, the 3 worm species have indirect life cycles. Seals become infected by ingesting crustaceans or benthic fish that serve as intermediate or paratenic hosts. *Corynosoma strumosum* inhabits the small intestine, *P. decipiens* the stomach and *O. circumlitus* the lung of its final host (Measures, 2001; Raga *et al*., 2009; Alonso-Farré *et al*., 2012). In spite of their ubiquity, several important knowledge gaps remain regarding the biology of these parasites, such as the extent of their tissue migration, when and how they derive nutrition during their life cycles and the degree by which they cause disease in their host. This is particularly the case for *O. circumlitus*, the lungworm which has been associated with significant respiratory disease if present in large numbers and/or concurrently with other helminthic or bacterial infections (Measures, 2001; Lehnert *et al*., 2010). Moreover its migratory route from the gut to the lungs following infection of the final host is still poorly understood, although it is suspected to be *via* the circulation (Barnett *et al*., 2019). In this study, stable isotope ratio analysis of seal parasites, host tissue and luminal content collected at the site of infection was employed to determine whether the technique can be used to explore further details of the biology and life style of these parasites.

## Materials and methods

### Sample collection and parasite identification

Parasites and tissue samples were collected during post-mortem examination of 4 stranded grey seals and 2 stranded harbour seals which had been brought to Seal Rescue Ireland and had subsequently died. Nasal mites were identified on the basis of morphology and anatomical location and all the collected specimens were used for stable isotope analysis. For Acanthocephala a subsample of whole worms was used for polymerase chain reaction (PCR) identification and the remainder for stable isotope ratio analysis. Nematodes were cut into 2 segments with one-third used for molecular identification and two-thirds for stable isotope analysis. For molecular identification, DNA was extracted using the QIAamp DNA Mini Kit following the manufacturer's instructions. Acanthocephala were identified based on a fragment of the small subunit ribosomal RNA gene sequence (ssrDNA) (Waeschenbach *et al*., 2007; Hermosilla *et al*., 2018), nematodes by amplification of the second internal transcribed spacer (ITS2) (Gasser *et al*., 1993). All primers and PCR conditions are provided in Table 2. Following Sanger sequencing in both the directions using the amplification primers, sequences were subjected to BLAST and aligned with each other and published sequences using Clustal Omega.

**Table 2.** Primers and PCR conditions used in the PCR analysis of nematodes and Acanthocephala

Target	Primers	PCR conditions	Reference
Second ITS2 region of nematodes	NC1 (forward): 5′-ACGTCTGGTTCAGGGTTGTT-3′ NC2 (reverse): 5′-TTAGTTTCTTTTCCTCCGCT-3′	95°C: 2 min; 40 cycles: 95°C: 1 min, 55°C: 1 min, 72°C: 1 min; 72°C: 5 min	Gasser *et al*. (1993)
ssrDNA of platyhelminths	Worm A (forward): 5′-GCGAATGGCTCATTAAATCAG-3′ Worm B (reverse): 5′-CTTGTTACGACTTTTACTTCC-3′	94°C: 2 min; 40 cycles: 94°C: 30 s, 54°C: 30 s, 72°C: 2 min; 72°C: 7 min	Waeschenbach *et al*. (2007); Hermosilla *et al*. (2018)

### Stable isotope ratio analysis

Samples subjected for bulk stable isotope ratio analysis included whole nasal mites, whole Acanthocephala specimens, the remaining two-thirds of nematode worms and duplicate samples of all tissues and (where available) luminal content at the anatomical sites where parasites were collected in each of the seals. Samples were individually freeze-dried, powdered using pestle and mortar and aliquoted into 1 mg portions in miniature tin capsules (Elemental Microanalysis D1028). They were then shipped to Iso-Analytical Limited, Crewe, UK for dual carbon and nitrogen isotope analysis by elemental analysis-isotope ratio mass spectrometry, using a Europa Scientific 20-20. The reference material used was IA-R068 (soy protein, *δ*^13^C_V−PDB_ = −25.22‰, *δ*^15^N_AIR_ = 0.99‰). IA-R069 (tuna protein, *δ*^13^C_V−PDB_ = −18.88‰, *δ*^15^N_AIR_ = 11.60‰) was run during the analysis of these samples as comparable quality control check samples, with a measurement precision (s.d., *n* = 12) of 0.03 and 0.04‰ for *δ*^13^C and *δ*^15^N, respectively.

Average *δ*^15^N and *δ*^13^C ± standard deviation (s.d.) were calculated for all seal tissues, luminal content and parasite species. The trophic discrimination factors Δ^15^N and Δ^13^C for each parasite–host sample combination was calculated by subtracting the *δ*^15^N or *δ*^13^C of the individual host tissue/luminal content collected from the same site where the parasite was found from the *δ*^15^N or *δ*13C value of individual parasites using the following formulas:
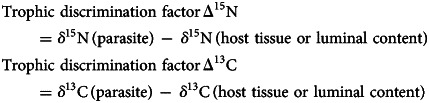


## Results

### Parasite identification

Based on their morphology and anatomical location, nasal mites were identified as *H. halichoeri*. PCR amplicons of all 7 Acanthocephala matched the published small subunit ribosomal RNA gene sequence of *C. strumosum* (e.g. OK096765). Out of a total of 16 analysed nematodes, 5 worms that had been collected from the stomach were identified as *P. decipiens*, based on 389 bp fragments (identical to HF680317). The remaining 12 worms produced 561 bp PCR amplicons which matched published sequences of *O. circumlitus* (e.g. KM458088). Of these, 4 were found in the lung, 5 in the stomach and 3 in the right ventricle of the heart and pulmonary artery.

### Stable isotope analysis

#### Nitrogen isotopic compositions and trophic discrimination factors Δ^15^N

**Figure 1.**
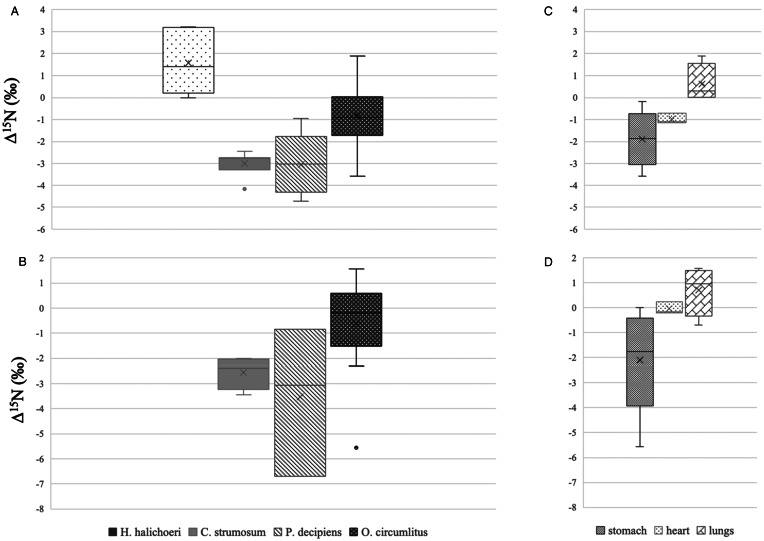
Δ^15^N (‰) of *Halarachne halichoeri*, *Corynosoma strumosum*, *Pseudoterranova decipiens* and *Otostrongylus circumlitus* in relation to (A) host tissue and (B) luminal content. Panels (C) and (D) show Δ^15^N (‰) of *O. circumlitus* worms located in the stomach, heart and lungs. ‘X’ indicates the mean, the middle line the median and the box the data between the 25th and 75th percentiles.

**Figure 2.**
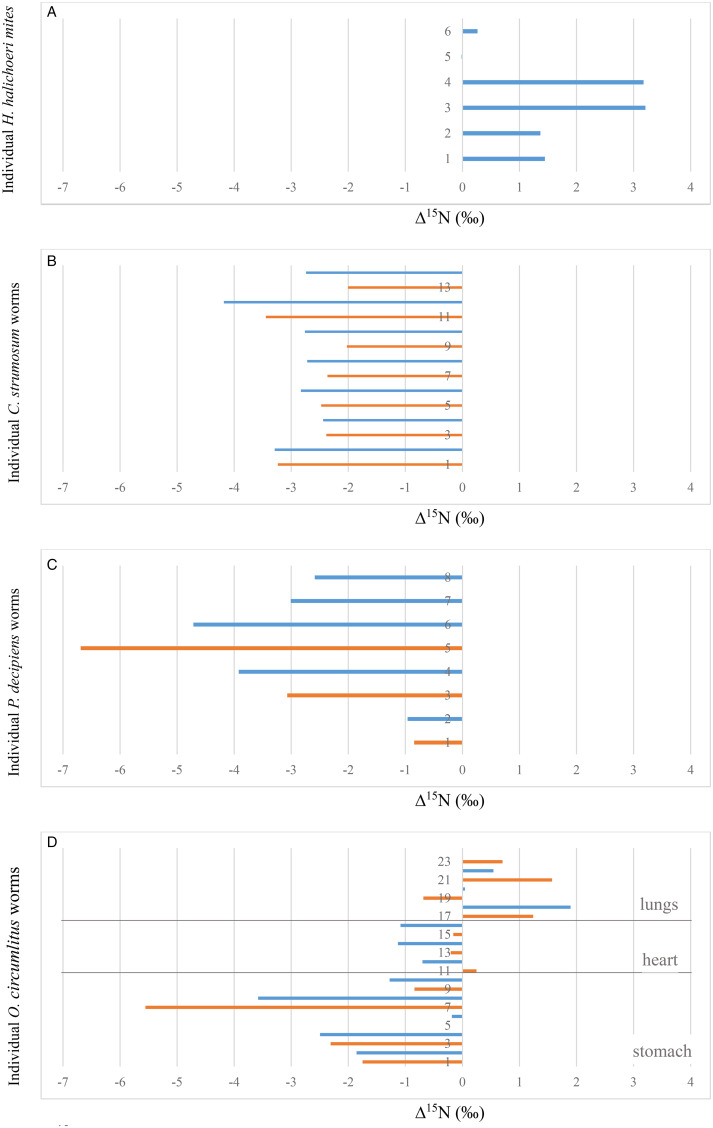
Δ^15^N (‰) of (A) *H. halichoeri*, (B) *C. strumosum*, (C) *P. decipiens* and (D) *O. circumlitus* in relation to host tissue (blue) and luminal content (orange). Numbers on the *Y*-axis refer to individual parasites.

A total of 6 mites and nasopharyngeal tissue samples from 4 seals (3 grey seals and 1 harbour seal) were subjected to stable isotope ratio analysis (in this case no luminal fluid was available). *δ*^15^N values of the mites measured 18.9‰ ± 1.4 s.d. on average, those of the seal nasal tissues were 17.3‰ ± 1.2 s.d. (Table 3). Δ^15^N, i.e. the difference in *δ*^15^N of the parasites and the individual host tissues was 1.6 ‰ ± 1.4 s.d. on average (Fig. 1), ranging between −0.015 and 3.2‰ (Fig. 2). For 5 out of 6 mites Δ^15^N was positive.

**Table 3.** *δ*^15^N and *δ*^13^C values (average ± s.d.) of (a) seal tissues, luminal content and (b) parasites

(a)					
	Nares (*n* = 4)	Stomach (*n* = 6)	Intestine (*n* = 3)	Lung (*n* = 5)	Heart (*n* = 1)
*δ*^15^N (‰)					
Tissue	17.3 ± 1.2	16.2 ± 1.2	16.7 ± 1.5	16.8 ± 1.5	16.1
Luminal content	n.d.	16.5 ± 1.9	16.3 ± 1.5	16.6 ± 1.1	15.2
*δ*^13^C (‰)					
Tissue	−17.1 ± 0.7	−18.1 ± 0.8	−17.3 ± 0.4	−17.7 ± 0.5	−18.9
Luminal content	n.d.	−17.6 ± 1.0	−18.5 ± 1.6	−17.7 ± 0.6	−17.6

(b)				
	*Halarachne halichoeri* (*n* = 6)	*Corynosoma strumosum* (*n* = 7)	*Pseudoterranova decipiens* (*n* = 5)	*Otostrongylus circumlitus* (*n* = 12)
*δ*^15^N (‰)	18.9 ± 1.4	13.7 ± 1.4	12.8 ± 0.8	15.8 ± 1.7
*δ*^13^C (‰)	−19.0 ± 0.9	−17.1 ± 0.9	−18.2 ± 1.3	−17.7 ± 1.1

Overall 7 Acanthocephalan worms, *C. strumosum* (all collected from grey seals) and 5 Anisakid nematodes, *P. decipiens* (collected from 3 grey seals and 1 harbour seal) were included in the study. Average *δ*^15^N values for *C. strumosum* were 13.7‰ ± 1.4 s.d., for *P. decipiens* 12.8‰ ± 0.8 s.d. Regarding the respective host tissues, *δ*^15^N for seal intestinal tissue was 16.7‰ ± 1.5 s.d. (luminal content: 16.3‰ ± 1.5 s.d.) and for the stomach tissue 16.2‰ ± 1.2 s.d. (luminal content: 16.5‰ ± 1.9 s.d.) respectively (Table 3). For both worm species, Δ^15^N was always negative with regards to host tissue and to luminal content. In the case of *C. strumosum* Δ^15^N ranged between −4.2 and −2.0‰ (average: −3.0‰ ± 0.6 s.d. with regards to host tissue; −2.6‰ ± 0.6 s.d. with regards to luminal content), in the case of *P. decipiens* between −6.7 and −0.8‰ (average −3.0‰ ± 1.4 s.d. with regards to host tissue; −3.5‰ ± 2.9 s.d. with regards to luminal content) (Figs 1 and 2).

Twelve *O. circumlitus* lungworms were collected from 3 grey and 2 harbour seals. *δ*^15^N for *O. circumlitus* measured 15.8‰ ± 1.7 s.d. on average. In this case, Δ^15^N varied with the location where the worms were found. For lungworms collected from the stomach Δ^15^N ranged between −5.6 and 0‰, for worms collected from the heart between −1.1 and 0.2‰, and for worms collected from the lungs, between −0.7 and 1.9‰ (Fig. 2). For all of the lungworms and tissues collected from the stomach Δ^15^N was negative (except for a single value which was 0), and all but 1 Δ^15^N value for lungworms collected from the heart were negative. In contrast, all worms in the lungs had a positive Δ^15^N with regards to the host tissue samples, and in all but 1 case also with regards to the luminal content.

#### Carbon isotopic compositions and trophic discrimination factors Δ^13^C

**Figure 3.**
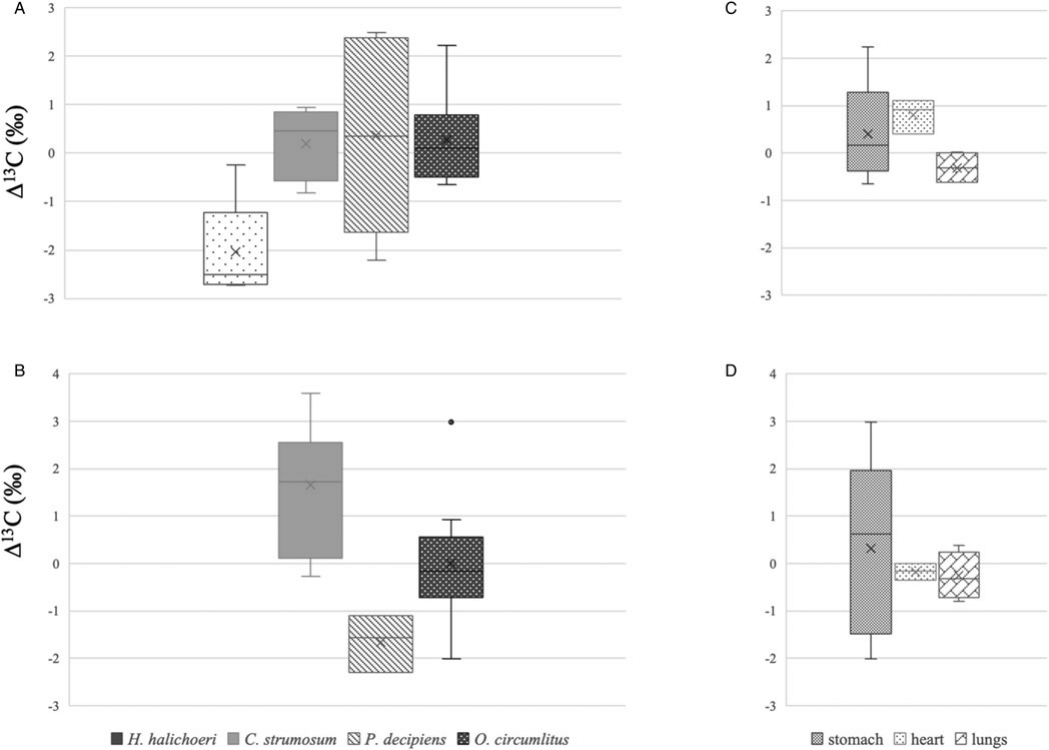
Δ^13^C (‰) of *H. halichoeri*, *C. strumosum*, *P. decipiens* and *O. circumlitus* in relation to (A) host tissue and (B) luminal content. Panels (C) and (D) show Δ^13^C (‰) of *O. circumlitus* worms located in the stomach, heart and lungs. ‘X’ indicates the mean, the middle line the median and the box the data between the 25th and 75th percentiles.

**Figure 4.**
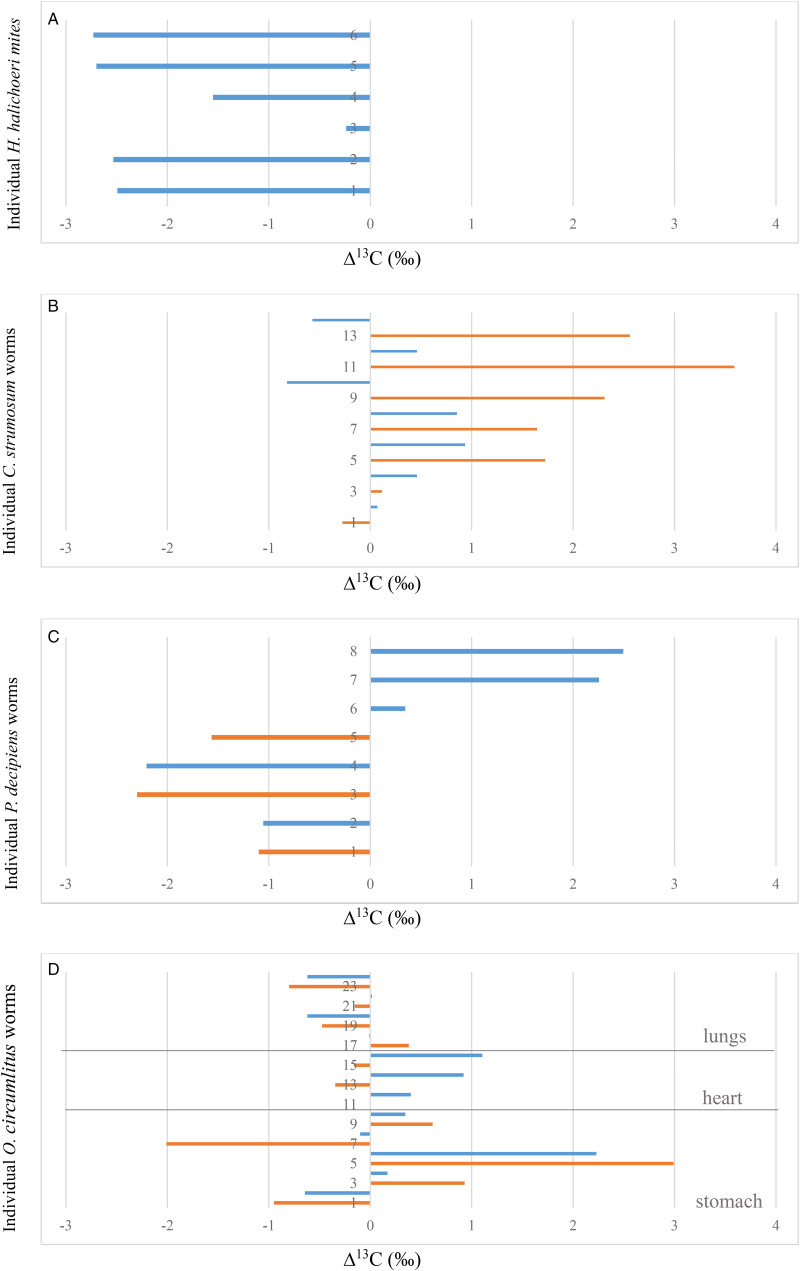
Δ^13^C (‰) of (A) *H. halichoeri*, (B) *C. strumosum*, (C) *P. decipiens* and (D) *O. circumlitus* in relation to host tissue (blue) and luminal content (orange). Numbers on the *Y*-axis refer to individual parasites.

Average *δ*^13^C values for all parasite species and seal tissues are also provided in Table 3. For *Halarachne halochoeri*, Δ^13^C, i.e. the difference in *δ*^13^C between nasal mites and individual host tissues ranged between −0.2 and −2.7‰ (average −2.0‰ ± 1.0 s.d.) (Fig. 3) and was negative for all 6 mites (Fig. 4). For *C. strumosum*, most Δ^13^C values were positive (average 0.2‰ ± 0.7 s.d.), while for *P. decipiens* most were negative (average 0.4‰ ± 2.0 s.d.) (Figs 3 and 4). For *O. circumlitus*, Δ^13^C again varied depending on where the worms were collected. While in this case the pattern was not as clear-cut as for Δ^15^N, there was a tendency for positive Δ^13^C values for worms found in the stomach and heart and negative Δ^13^C values for worms in the lungs (Fig. 4). The difference in *δ*^13^C between parasite and host tissue was greatest for worms in the stomach (overall average 0.3‰ ± 0.8 s.d.).

Biplots of *δ*^13^C vs *δ*^15^N values for host tissues, luminal content and the various parasite species are provided as Supplementary materials (Figs S1 and S2).

## Discussion

*Halarachne halochoeri* mites inhabit the upper respiratory tract, particularly the nasal passages and nasopharyngeal mucosa of seals. They are transmitted directly *via* sneezing or direct nasal contact when seals congregate on land (Fay and Furman, 1982; Raga *et al*., 2009; Alonso-Farré *et al*., 2012). Of all parasites investigated in this study, nasal mites were the only group that consistently recorded positive Δ^15^N values with regards to their host's nasopharyngeal tissues, with 2 out of 6 mites exceeding Δ^15^N values of 3.1‰. This trophic relationship indicates a classical predator–prey relationship, confirming previous observations that *H. halochoeri*, similar to other parasitic mites, feeds directly on host tissue (Alonso-Farré *et al*., 2012). Similar Δ^15^N values have been reported for other ectoparasites including ixodid ticks (Schmidt *et al*., 2011; Heylen *et al*., 2019) and parasitic copepods (Kamiya *et al*., 2020). In contrast Δ^13^C values were negative (with 4 out of 6 mites measuring −2.5‰ or less). Reduced or close to zero Δ^13^C values have also been reported in ticks and parasitic copepods relative to their host tissues (Schmidt *et al*., 2011; Heylen *et al*., 2019; Kamiya *et al*., 2020), although none were quite as low as those recorded here. It has been argued that parasites often have low *δ*^13^C values because many utilize host-derived lipids and fatty acids directly instead of synthesizing them *de novo* (Riekenberg *et al*., 2021), and these host-derived lipids are typically depleted in heavier isotopes because, for kinetic reasons, lighter isotopes are favoured during lipid synthesis (Fagan *et al*., 2011; Nachev *et al*., 2017). It is also important to point out that we compared *δ*^13^C values from whole mites (containing a range of carbon-rich metabolites, storage and structural elements) with samples of the hosts’ nasopharyngeal mucosa, which would have been more homogenous and presumably contained fewer lipid stores. This was indicated by the C:N ratios of the 2 sample types (which measured 5.9 ± 0.4 s.d. for *H. halochoeri* and 3.6 ± 0.3 s.d. for seal nasopharyngeal tissue on average, data not shown). No lipid removal step was included in our analysis. Another factor that may play a part in the metabolism of ectoparasites is their endosymbionts. In the case of ticks, symbionts are known to serve as sources of vitamins and energy, affecting many aspects of the tick's biology and life cycle (Bonnet *et al*., 2017). It is possible that parasitic mites have similar symbiotic relationships which may affect their lipid biosynthesis pathways and isotope composition.

Seals become infected with the Acanthocephalan or thorny-headed worm, *C. strumosum*, by ingesting infected nearshore amphipod intermediate hosts or demersal fish such as cod or herring which can act as paratenic hosts (Nickol *et al*., 2002; Raga *et al*., 2009). Inside the small intestine of seals which serve as final hosts, the adult worms attach to the mucosa using an array of hooks on their retractable proboscis. In contrast to parasitic mites, which due to their exoskeleton and anatomical location are unable to absorb nutrients directly *via* their body surface, Acanthocephala resemble cestodes in that they lack a mouth and digestive system and absorb all nutrients *via* their body surface. This nutrient absorption is facilitated by a specialized tegument consisting of a complex syncytium with a lacunar transport system of interconnecting canals and an internal lipid storage system (Deplazes *et al*., 2016). Interestingly, Δ^15^N and Δ^13^C values measured for *C. strumosum* were exactly the opposite to those recorded for *H. halochoeri*, i.e. they were strongly negative with regards to Δ^15^N and mostly positive with regards to Δ^13^C. This was true in relation to the hosts’ intestinal tissue as well as the luminal content. Negative Δ^15^N values have also been reported for Acanthocephala species that infect fish as final hosts (Nachev *et al*., 2017; Kamiya *et al*., 2020). Strong depletion in *δ*^15^N indicates that rather than absorbing half-digested food in the hosts’ intestine indiscriminately, which would place them on the same trophic level as the host, Acanthocephala selectively absorb isotopically lighter host- or host microbiome-derived molecules (Nachev *et al*., 2017), possibly facilitated by the specialized tegumental syncytium. Alternatively *δ*^15^N depletion in adult *C. strumosum* may be due to peptides and amino acids absorbed by previous life stage in the intermediate or paratenic host, amphipods or fish, which themselves would have been on a lower trophic level than the final seal host. The comparatively high Δ^13^C values observed in the present study are puzzling. These compare to more varied but much lower Δ^13^C values reported in Acanthocephalan parasites in fish ranging from negative to close to zero (Nachev *et al*., 2017; Kamiya *et al*., 2020). Generally, *δ*^13^C enrichment resulting in Δ^13^C values of 0.5–1‰ are thought to indicate direct ingestion of host tissues. Four of the 7 worms analysed in this study were in this bracket with regards to the host's intestinal tissues, 2 were negative (−0.8 to −0.6‰), while one was close to zero. Even higher positive Δ^13^C values, ranging up to 3.6‰, were recorded in relation to the intestinal content, suggesting either specialized use of a fraction of intestinal content or selective use of protein- or carbohydrate-derived materials from the host tissues. It is also possible that the *δ*^13^C values of at least some of the worms reflected lipids absorbed from the previous host. The C:N ratio of the parasites was very similar to that of the hosts’ intestinal tissues (3.7 ± 0.1 s.d. for *C. strumosum* and 3.5 ± 0.2 s.d. for the seal tissues, respectively).

Both, *P. decipiens* and *O. circumlitus* have indirect life cycles. The larvae of the anisakid nematode, *P. decipiens*, undergo development in the gut and haemocoel of benthic crustaceans and macroinvertebrates, before infecting benthophagous fish, where they migrate to somatic muscle tissue (McClelland, 2002). Seals become infected by predation on these smaller fish or on larger demersal fish that have ingested them. In the final host, the adult worms attach to the stomach wall (mostly in the fundus) with their heads embedded in the tissues. Regarding the lungworm, *O. circumlitus*, the life cycle is not yet fully known. While it is thought to largely resemble that of *P. decipiens*, an invertebrate host may not be required (Lehnert *et al*., 2010). Larval stages have been detected in the mucosa, mesentery and pancreas of turbot which are thought to serve as intermediate hosts. It has been suggested that once the worm has been ingested by the final seal host, it migrates to the lungs *via* the hepatic portal system, heart and pulmonary circulation (Measures, 2001; Barnett *et al*., 2019). However, the fact that we detected quite a number of *O. circumlitus* worms in the stomach (with identities confirmed by PCR) indicates that the parasite might spend a considerable amount of time in the stomach before embarking on its tissue migration.

The Δ^15^N values of both nematode species were strongly negative when they were collected from the seals’ stomach, resembling those of *C. strumosum*. Whether this means that they derive their nutrition in a similar way remains to be seen. While the nematode cuticle would preclude the worms from absorbing nutrients across the body surface, nematodes with very small buccal capsules such as *P. decipiens* and *O. circumlitus* are thought to feed on mucosal fluid, products of host digestion and cell debris (Taylor *et al*., 2016*a*). Consequently low *δ*^15^N values in these worms may be due to selective absorption of *δ*^15^N-depleted host metabolites. Alternatively they may be a reflection of nutrients absorbed from previous hosts by earlier life stages. Again, Δ^13^C values were more variable and inconsistent, with some highly positive and some highly negative values recorded for both stomach tissue and contents. The C:N ratio for both nematode species was also quite high (5.5 ± 1.4 s.d. for *P. decipiens* and 5.0 ± 1.0 s.d. *O. circumlitus*) possibly as a result of carbon-rich energy stores in the form of glycogen (accounting for about 10% of the dry weight of the worm) and chitin in the cuticle.

What was interesting was the shift in *δ*^15^N values in *O. circumlitus* worms collected from other anatomical sites. As the worms migrated from the stomach to the heart and lungs their *δ*^15^N ratios increased leading to less negative Δ^15^N values in relation to heart tissue and mostly positive Δ^15^N values in relation to lung tissue (ranging from 0.05 to 1.9‰). This implies a gradual shift to a higher trophic level and an adaptation to a more predatory life style associated with the more pathogenic nature of *O. circumlitus* compared to that of the other worm species. The fact that Δ^15^N did not quite reach the threshold of 3.4‰, considered the hallmark of a predator–prey relationship, may be due to the fact that increased protein quality in the diet of a top predator, such as a seal, can result in a smaller ‰ difference between the diet and the consumer (Riekenberg *et al*., 2021). It is also interesting to note that the gradual shift of *δ*^15^N values of *O. circumlitus* worms in the various tissues indicates that the parasites in the stomach were not flushed there from the lungs *via* the trachea just before or after death, but had apparently been there for a significant period of time. Whether this delayed migration is part of the normal life cycle or only occurs during very heavy infestations (as has been reported for other histiotrophic parasites) remains to be determined. Δ^13^C values were mostly negatively correlated with Δ^15^N values, with positive ratios in the heart and negative ones in the lungs.

In conclusion, this is the first study, to our knowledge, that investigates the stable isotope composition of common seal parasites. Based on Δ^15^N values the parasite–host associations could be clearly divided into those that resembled a trophic predator–prey relationship (*H. halochoeri* in the nasopharyngeal passages and *O. circumlitus* in the lungs) and those that did not (*C. strumosum* in the intestine, and *P. decipiens* and *O. circumlitus* in the stomach). It is tempting to suggest that Δ^15^N values are indicative of the level of pathogenicity associated with a parasite. Parasites with positive Δ^15^N values that feed directly on host tissue would be expected to be more invasive and more likely to cause tissue damage than parasites that absorb host molecules. As a result they would be expected to affect their hosts’ health more severely, both directly and indirectly by eliciting host responses which may contribute to their pathogenicity. It may be worthwhile to re-examine the potential pathogenicity of nasal mites in seals, especially considering the fact that they can cause severe clinical signs in cats and dogs if present in large numbers (Taylor *et al*., 2016*b*).

In contrast to Δ^15^N, Δ^13^C values were much more difficult to interpret. This was probably at least partly due to the fact that we compared parasite bulk samples containing a whole complement of organs and tissues to relatively homogenous seal tissue samples. Finally there were no obvious benefits to including luminal content in addition to host tissue samples in the analysis.

## Supporting information

Zintl et al. supplementary material Zintl et al. supplementary material

## Data Availability

All data generated during this study are included in the published article. The datasets are available on request from the corresponding author.'

## Supplementary material

The supplementary material for this article can be found at https://doi.org/10.1017/S003118202400074X

## References

[ref1] Alonso-Farré JM, D'Silva JI and Gestal C (2012) Nasopharyngeal mites *Halarachne halichoeri* (Allman, 1847) in grey seals stranded on the NW Spanish Atlantic Coast. Veterinary Parasitology 183, 317–322. 21871735 10.1016/j.vetpar.2011.08.002

[ref2] Barnett JEF, Bexton S, Fraija-Fernández N, Chooneea D and Wessels ME (2019) Novel pulmonary vasculitis with plendore-Hoeppli reaction in grey seals (*Halichoerus grypus*) associated with *Otostrongylus circumlitus* infection. Journal of Comparative Pathology 173, 83–91. 31812177 10.1016/j.jcpa.2019.10.009

[ref3] Bonnet SI, Binetruy F, Hernández-Jarguín AM and Duron O (2017) The tick microbiome: why non-pathogenic microorganisms matter in tick biology and pathogen transmission. Frontiers in Cellular and Infection Microbiology 7, 236. 28642842 10.3389/fcimb.2017.00236 PMC5462901

[ref4] Caut S, Angulo E and Courchamp F (2009). Variation in discrimination factors (Δ^15^N and Δ^13^C): the effect of diet isotopic values and applications for diet reconstruction. Journal of Applied Ecology 46, 443–453.

[ref5] Deplazes P, Eckert J, Mathis A, von Samson-Himmelstjerna G and Zahner H (2016). Parasitology in Veterinary Medicine. The Netherlands: Wageningen Academic Publishers, p. 384.

[ref6] Fagan K-A, Koops MA, Arts MT and Power M (2011). Assessing the utility of C:N ratios for predicting lipid content in fishes. Canadian Journal of Fisheries and Aquatic Sciences 68, 374–385.

[ref7] Fay FH and Furman DP (1982). Nasal mites (Acari: Halarachnidae) in the spotted seal, *Phoca largha* Pallas, and other pinnipeds of Alaskan waters. Journal of Wildlife Diseases 18, 63–68. 7097872 10.7589/0090-3558-18.1.63

[ref8] Gasser RB, Chilton NB, Hoste H and Beveridge I (1993) Rapid sequencing of rDNA from single worms and eggs of parasitic helminths. Nucleic Acids Research 21, 2525–2526. 8506152 10.1093/nar/21.10.2525 PMC309567

[ref9] Hermosilla C, Hirzmann J, Silva LM, Scheufen S, Prenger-Berninghoff E, Ewers C, Häussermann V, Försterra G, Poppert S and Taubert A (2018) Gastrointestinal parasites and bacteria in free-living South American sea lions (*Otaria flavescens*) in Chilean Comau fjord and new host record of a *Diphyllobothrium scoticum*-like cestode. Frontiers in Marine Science 5, 259.

[ref10] Heylen D, Schmidt O, Dautel H, Gern L, Kampen H, Newton J and Gray J (2019) Host identification in unfed ticks from stable isotope compositions (*δ*^13^C and *δ*^15^ N). Medical and Veterinary Entomology 33, 360–366. 30883848 10.1111/mve.12372

[ref11] Kamiya E, Urabe M and Okuda N (2020) Does atypical ^15^N and ^13^C enrichment in parasites result from isotope ratio variation of host tissues they are infected? Limnology 21, 139–149.

[ref12] Lehnert K, von Samson-Himmelstjerna G, Schaudien D, Bleidorn C, Wohlsein P and Siebert U (2010) Transmission of lungworms of harbour porpoises and harbour seals: molecular tools determine potential vertebrate intermediate hosts. International Journal for Parasitology 40, 845–853. 20123100 10.1016/j.ijpara.2009.12.008

[ref13] McClelland G (2002) The trouble with sealworms (*Pseudoterranova decipiens* species complex, Nematoda): a review. Parasitology 124, S183–S203. 12396224 10.1017/s0031182002001658

[ref14] Measures LN (2001) Chapter 10: Lungworms of marine mammals. In Samuel WM, Pybus JM and Kocan A (eds), Parasitic Diseases of Wild Mammals. Wiley Online Library, pp. 279–300.

[ref15] Nachev M, Jochmann MA, Walter F, Wolbert JB, Schulte SM, Schmidt TC and Sures B (2017). Understanding trophic interactions in host–parasite associations using stable isotopes of carbon and nitrogen. Parasites & Vectors 10, 1–9. 28212669 10.1186/s13071-017-2030-y PMC5316170

[ref16] Nickol BB, Helle E and Valtonen ET (2002). *Corynosoma magdalen*i in grey seals from the Gulf of Bothnia, with emended descriptions of *Corynosoma strumosum* and *Corynosoma magdaleni*. The Journal of Parasitology 88, 1222–1229. 12537117 10.1645/0022-3395(2002)088[1222:CMIGSF]2.0.CO;2

[ref17] Post DM (2002) Using stable isotopes to estimate trophic position: models, methods, and assumptions. Ecology 83, 703–718.

[ref18] Raga JA, Fernández M, Balbuena JA and Aznar FJ (2009) Parasites. In Perrin WF, Würsig B and Thewissen JGM (eds), Encyclopedia of Marine Mammals, 2nd Edn. Academic Press, pp. 821–830.

[ref19] Riekenberg PM, Briand MJ, Moléana T, Sasal P, van der Meer MT, Thieltges DW and Letourneur Y (2021). Isotopic discrimination in helminths infecting coral reef fishes depends on parasite group, habitat within host, and host stable isotope value. Scientific Reports 11, 4638. 33633261 10.1038/s41598-021-84255-0 PMC7907083

[ref20] Schmidt O, Dautel H, Newton J and Gray JS (2011). Natural isotope signatures of host blood are replicated in moulted ticks. Ticks and Tick-borne Diseases 2, 225–227. 22108017 10.1016/j.ttbdis.2011.09.006

[ref21] Taylor MA, Coop RL and Wall RL (2016a) Chapter 1, Veterinary helminthology. In Veterinary Parasitology, 4th Edn. West Sussex, UK: Wiley Blackwell, pp. 1–109.

[ref22] Taylor MA, Coop RL and Wall RL (2016b) Chapter 12, Parasites of cats and dogs. In Veterinary Parasitology, 4th Edn. West Sussex, UK: Wiley Blackwell, pp. 599–677.

[ref23] Thieltges DW, Goedknegt MA, O'Dwyer K, Senior AM and Kamiya T (2019) Parasites and stable isotopes: a comparative analysis of isotopic discrimination in parasitic trophic interactions. Oikos 128, 1329–1339.

[ref24] Waeschenbach A, Webster BL, Bray RA and Littlewood DTJ (2007) Added resolution among ordinal level relationships of tapeworms (Platyhelminthes: Cestoda) with complete small and large subunit nuclear ribosomal RNA genes. Molecular Phylogenetics and Evolution 45, 311–325. 17485227 10.1016/j.ympev.2007.03.019

