# Supplementary material for: Use of stable isotope ratio analysis to investigate the biology and clinical significance of seal parasites
Source: Parasitology. 2024 Sep 25;151(7):744–52. doi: 10.1017/S003118202400074X (PMC11474010; doi:10.1017/S003118202400074X)
Supplement: Zintl et al. supplementary material [file S003118202400074Xsup001.docx]

Intestinal tissue

Nasal tissue

Lung tissue

Stomach tissue

Lung content

Stomach content

Intestinal content

*O. circumlitus*

*P. decipiens*

*C. strumosum*

*H. halichoeri*

Harbour seal

Harbour seal

δ^13^C (‰)

δ^13^C (‰)

δ^13^C (‰)

δ^13^C (‰)

δ^13^C (‰)

Grey seal

Grey seal

δ^15^N (‰)

δ^15^N (‰)

δ^15^N (‰)

δ^15^N (‰)

δ^15^N (‰)

δ^15^N (‰)

δ^13^C (‰)

Grey seal

Grey seal

**Figure 1S** Biplots of δ^13^C versus δ^15^N values for host tissues, luminal content, *O. circumlitus*, *P. decipiens*, *C. strumosum* and *H. halichoeri* species in the 6 seals investigated during the study (including 4 Grey and 2 Harbour seals)

Grey seals (n=4)

Harbour seals (n=2)

Intestinal tissue

Nasal tissue

Lung tissue

Stomach tissue

Lung content

Stomach content

Intestinal content

*O. circumlitus*

*P. decipiens*

*C. strumosum*

*H. halichoeri*

**Figure 2S** Biplots of mean δ^13^C versus mean δ^15^N (± s.d.) for host tissues, luminal content, *O. circumlitus*, *P. decipiens*, *C. strumosum* and *H. halichoeri* species in the 6 seals investigated during the study (including 4 Grey and 2 Harbour seals)
